# Microgravity Therapy as Treatment for Decelerated Aging and Successful Longevity

**DOI:** 10.3390/ijms26136544

**Published:** 2025-07-07

**Authors:** Nadine Mozalbat, Lital Sharvit, Gil Atzmon

**Affiliations:** Medical School, Department of Human Biology, Faculty of Natural Sciences, University of Haifa, Haifa 3498838, Israel; nadine_mo@hotmail.com (N.M.); lsharvit@univ.haifa.ac.il (L.S.)

**Keywords:** aging, hallmarks of aging, longevity, microgravity, NASA’s twins study, spaceflight, cellular senescence

## Abstract

Aging is a complex biological process marked by a progressive decline in cellular function, leading to age-related diseases such as neurodegenerative disorders, cancer, and cardiovascular diseases. Despite significant advancements in aging research, finding effective interventions to decelerate aging remains a challenge. This review explores microgravity as a novel therapeutic approach to combat aging and promote healthy longevity. The hallmarks of aging, including genomic instability, telomere shortening, and cellular senescence, form the basis for understanding the molecular mechanisms behind aging. Interestingly, microgravity has been shown to accelerate aging-like processes in model organisms and human tissues, making it an ideal environment for studying aging mechanisms in an accelerated manner. Spaceflight studies, such as NASA’s Twins Study and experiments aboard the International Space Station (ISS), reveal striking parallels between the physiological changes induced by microgravity and those observed in aging populations, including muscle atrophy, bone density loss, cardiovascular deconditioning, and immune system decline in a microgravity environment. However, upon microgravity recovery, cellular behavior, gene expression, and tissue regeneration were seen, providing vital insights into aging mechanisms and prospective therapeutic approaches. This review examines the potential of microgravity-based technologies to pioneer novel strategies for decelerating aging and enhancing healthspan under natural gravity, paving the way for breakthroughs in longevity therapies.

## 1. Introduction

### 1.1. Understanding Aging: A Foundation for Longevity Research

Aging is marked by a progressive decline in physiological function, which reduces resilience and increases the risk of mortality [1,2]. This fundamental biological process involves complex molecular and cellular changes, including a diminished capacity to manage cellular stress responses, leading to mitochondrial dysfunction [3], increased production of reactive oxygen species (ROS) [4], DNA damage [5], telomere shortening [6], and impaired processing of microRNAs [7]. These cellular changes contribute to the emergence of various age-related diseases, such as neurodegenerative disorders [8], cancer [9], diabetes [10], and hypertension [11]. Although aging is a natural and universal process, its biological consequences vary. While many individuals remain healthy and independent, others face physiological decline and increased disease risk. According to the World Health Organization, the number of people aged 60 and above is expected to reach 2.1 billion by 2050, with over 80% of them living in developing countries [12]. This demographic shift is expected to increase the global burden of age-associated diseases and highlights the need for effective interventions. A comprehensive understanding of aging requires examining its hallmarks, including core biological processes that collectively drive this complex phenomenon. These hallmarks were first proposed by López-Otín et al. [13] and have been recently updated [1]. Unraveling these mechanisms is critical for identifying therapeutic strategies to promote healthspan and combat age-related diseases. Given the global rise in aging populations, advancing research in this field is essential for addressing the growing societal and healthcare demands. This review explores the fundamental mechanisms of aging and highlights common treatments, and offers microgravity as a novel approach to advancing aging and longevity research.

### 1.2. The Hallmarks of Aging

**Figure 1:**
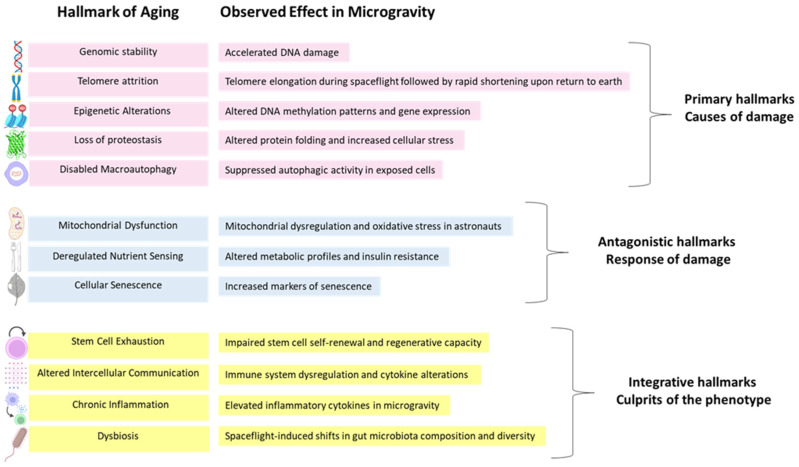
Hallmarks of aging and corresponding effects observed in microgravity. The twelve hallmarks of aging are categorized into three functional groups: primary (pink), antagonistic (blue), and integrative (yellow). Each hallmark is represented by an icon and label, accompanied by a brief summary of reported effects under microgravity. The figure highlights the potential of microgravity as a model to investigate fundamental mechanisms of aging.

The hallmarks of aging encompass fundamental biological processes that collectively drive the aging process. Currently, twelve hallmarks of aging have been defined [1]. At the core of these processes is genomic instability, where accumulated DNA damage impairs cellular integrity, initiating increased susceptibility to age-related diseases. This instability is compounded by telomere attrition, as the progressive shortening of chromosome ends eventually halts cell division and accelerates cellular aging [6]. Beyond genetic factors, epigenetic alterations disrupt normal gene expression patterns, leading to dysregulated cellular dysfunction. Simultaneously, the loss of proteostasis impairs the cell’s ability to maintain protein structure and function, resulting in protein misfolding, aggregation, and cellular stress [14]. A decline in macroautophagy, a critical cellular degradation pathway, further contributes to proteostasis failure. Mitochondrial dysfunction exacerbates these effects by reducing energy production and increasing oxidative stress, accelerating cellular damage. As a protective response, some cells enter cellular senescence, a state of permanent growth arrest that initially prevents the propagation of damaged cells. However, senescent cells accumulate over time and secrete inflammatory factors; senescence-associated secretory phenotype (SASP), contributing to chronic tissue dysfunction [15,16]. Stem cell exhaustion further impairs tissue regeneration, leading to a decline in organ function. Additionally, altered intercellular communication disrupts signaling pathways essential for tissue homeostasis, often promoting chronic inflammation, which further accelerates aging-related deterioration. Finally, dysbiosis, characterized by age-associated shifts in the gut microbiome, affects immune function, metabolism, and systemic health (reviewed in [1]). Together, these hallmarks represent interconnected mechanisms that contribute to the progressive decline of physiological function, ultimately leading to age-related diseases. Understanding these processes provides a foundation for developing therapeutic strategies to promote healthy aging and longevity (Figure 1).

### 1.3. Aging Associated Diseases

Aging is a major risk factor for the onset and progression of numerous chronic diseases, which, in turn, exacerbate aging itself, posing significant challenges to healthspan and quality of life. As physiological systems deteriorate over time, molecular and cellular dysfunctions accumulate, leading to age-related pathologies such as neurodegenerative disorders, metabolic syndromes, cardiovascular diseases (CVD), and cancer [17,18]. Among these, neurodegenerative diseases are particularly prevalent, with aging serving as their primary risk factor. Alzheimer’s disease (AD), the most common neurodegenerative disorder worldwide, demonstrates a strong correlation with advancing age, as its incidence rises significantly in elderly populations [19]. Similarly, aging profoundly impacts the cardiovascular system, contributing to an increased prevalence of cardiovascular diseases (CVD), such as atherosclerosis, hypertension, myocardial infarction, and stroke [20]. As life expectancy rises, the burden of CVD is projected to grow, with estimates suggesting that by 2030, approximately 20% of the global population will be over 65 years old, and CVD will account for 40% of all deaths [21]. The immune system also undergoes significant age-related changes, leading to immunosenescence, a decline in immune function that increases susceptibility to infections, reduces the body’s ability to combat cancer, and elevates the risk of autoimmune diseases [22,23]. Notably, while cancer is a major age-associated pathology, its occurrence is not inevitable. It affects about one-third of women and half of men in their lifetime, suggesting that genetic predisposition, lifestyle factors, and stochastic processes contribute to its development [24]. Despite this variability, aging remains the strongest risk factor for cancer, with incidence peaking around 85 years [25]. Interestingly, beyond age 90, both cancer incidence and mortality decline, and by age 100, cancer accounts for less than 5% of overall morbidity and mortality. In contrast, respiratory, infectious, and neurodegenerative diseases become increasingly prevalent in centenarians, highlighting a shift in disease burden with extreme longevity [26,27].

Given the significant influence of aging on disease susceptibility, elucidating the complex relationship between aging and associated pathologies is essential for the development of targeted interventions. A deeper understanding of the molecular and physiological mechanisms underlying these conditions is pivotal for the creation of therapeutic strategies aimed at alleviating age-related health burdens and extending healthspan. Consequently, a variety of pharmacological and non-pharmacological treatments are being explored to address these challenges and promote longevity.

### 1.4. Major Aging Treatments

Advancements in aging research have led to the exploration of various therapeutic approaches aimed at delaying age-related decline and extending healthspan. Among these, pharmacological interventions such as rapamycin, an mTOR inhibitor, have shown promise in promoting longevity and enhancing cellular resilience. Studies have demonstrated that rapamycin can inhibit the mechanistic target of rapamycin (mTOR) protein kinase, leading to extended lifespan in model organisms such as yeast, *C. elegans*, and fruit flies [28]. Over the past decade, there has been a marked increase in studies investigating rapamycin’s effects on physiological functions and disease processes in mammals. These studies have demonstrated that rapamycin can extend both lifespan and healthspan in mice, even when treatment is initiated later in life. Its benefits span multiple systems, including improvements in cardiac performance, immune regulation, and reductions in cellular senescence, supporting its potential for translation into human therapies targeting age-related diseases [29].

Other than rapamycin, senolytics have emerged as a novel class of drugs designed to selectively eliminate senescent cells, which accumulate with age and contribute to chronic inflammation and tissue dysfunction [30,31]. Senescent cells evade apoptosis through the upregulation of several anti-apoptotic regulators, including dependence receptors, PI3K/Akt, and BCL-2, making them viable targets for therapeutic intervention [32]. Recent studies have further demonstrated the potential of senolytic agents such as dasatinib and quercetin in effectively clearing senescent cells in both animal models and early-stage human trials. In preclinical research, their administration has led to reduced senescence markers and improved tissue function in models of intervertebral disk degeneration, osteoarthritis, and pulmonary inflammation. Complementary findings from pilot clinical trials in older adults indicate that dasatinib and quercetin are safe and may improve physical and cognitive performance [33,34,35,36].

Furthermore, widely used compounds such as metformin and resveratrol are being investigated for their ability to modulate aging-associated metabolic and inflammatory pathways. Metformin has been shown to improve mitochondrial function and reduce oxidative stress and inflammation [37], while resveratrol exerts antioxidant effects and enhances the expression of longevity-associated genes such as SIRT1 and CAT [38].

In addition to pharmacological treatments, non-pharmacological strategies, including caloric restriction and regular physical exercise, have been shown to enhance metabolic health, reduce inflammation, and improve mitochondrial function [39]. Alongside pharmacological and non-pharmacological strategies, biologics and biomaterials are also being explored as potential approaches to mitigate age-related decline. Biologics such as stem cell therapies aim to support tissue regeneration and immune modulation, offering promising avenues for reversing age-associated functional deterioration [40]. Monoclonal antibodies targeting senescent cells and age-related molecular pathways have also emerged as experimental strategies to delay or prevent aging-related dysfunction [41]. Furthermore, biomaterials, such as scaffolds and injectable matrices, are increasingly being utilized in tissue engineering to promote the repair and structural support of aged or damaged tissues [42]. These interventions, summarized in Table 1, alongside ongoing research into novel therapeutic targets, offer promising avenues for promoting longevity and reducing the public health burden of age-related diseases.

### 1.5. Microgravity’s Impact on Human Physiology

Given the growing aging population, understanding the mechanisms driving the decline in bodily functions with age has become increasingly essential. Identifying strategies to slow down or even prevent these changes could enhance public health and extend longevity, while yielding significant economic benefits for society [43]. This task is complicated by the long follow-up timeframes needed for such studies, even short-lived rodent models take about three years to observe lifespan changes, and studies in primates can last anywhere from 15 to 30 years [44]. The need for a short-duration human aging model is challenging, and decades of research have not generated one. Recently, we proposed a model that may overcome these challenges. Using pregnancy as a model for aging, we offered a human model that can be used for studying the aging process [45]. Yet, this model, although it solves most of the weaknesses of the current models, has its own drawbacks [46]. Gravity plays a crucial role in shaping human physiology, and prolonged exposure to microgravity during space missions can lead to various pathologies that mirror age-related changes. Astronauts frequently experience significant bone density loss [47], muscle atrophy [48], cardiovascular deconditioning [49], immunological [50], cerebrovascular [51], cognitive alterations [36], and metabolic problems [50,52]. These changes, observed in both aging populations and astronauts in microgravity, reveal striking similarities that highlight the potential of utilizing space as a model for accelerated aging research. In microgravity, aging-like processes are accelerated by up to ten times, occurring over days or weeks rather than years. This makes the space environment a unique model for studying aging in an accelerated format, offering insights that are otherwise unattainable on Earth [53].

### 1.6. NASA’s Twins Study

NASA’s landmark Twins Study provided a unique framework for studying the effects of microgravity on aging. This year-long experiment followed a pair of genetically identical twin astronauts, with one spending 340 days aboard the International Space Station (ISS), while the other remained on Earth. The extended duration of this mission allowed researchers to collect comprehensive multi-omic, physiological, and cognitive data before, during, and after the flight, providing rare longitudinal insights into human adaptation to space. Upon the space traveler’s return, comparative analysis revealed notable differences in physiological, biological, and functional characteristics, with the Earth-bound twin generally exhibiting healthier parameters. Although the mechanisms remain unclear, factors like astronauts’ healthy lifestyle, weight changes, or shifts in cell populations with longer telomeres may play a role [52].

Key observations from the NASA Twins Study align with several hallmarks of aging. For instance, telomere length dynamics were significantly altered during the mission, with elongation observed in space followed by shortening upon return, reflecting fluctuations relevant to telomere attrition. Additionally, gene expression patterns were widely affected, especially in pathways associated with immune response, DNA repair, and oxidative stress, indicating shifts tied to epigenetic alterations and genomic instability [52]. The spaceflight-induced immune dysregulation and elevated inflammation levels further correspond to altered intercellular communication, a key feature of aging-related chronic inflammation [52]. Notably, returning to Earth gravity also activated muscle repair and regeneration pathways, suggesting a complex impact of space travel on aging processes [50,52]. Together, these findings illustrate how microgravity conditions can accelerate or modify aging-related processes in a human model and support the relevance of space-based platforms for aging research.

### 1.7. Candidate Genes Regulated by Microgravity

Gene regulation is a key biological mechanism that enables cells and organisms to sustain life and adapt to environmental changes. Microgravity induces substantial transcriptomic remodeling in human cells, impacting pathways involved in DNA repair, chromatin remodeling, cytoskeletal organization, mitochondrial function, and cellular stress responses. These pathways closely align with the hallmarks of aging. Identifying genes consistently regulated under microgravity is essential for understanding cellular adaptation and for developing therapeutic strategies to mitigate microgravity-induced and aging-like dysfunction.

Transcriptomic analyses of human cell lines exposed to both real and simulated microgravity have identified a panel of eleven candidate genes exhibiting consistent differential expression. Upregulated genes include CSGALNACT2, CSNK2A2, HIPK1, MBNL2, PHF21A, and RAP1A, which are involved in pathways such as glycosaminoglycan biosynthesis, chromatin remodeling, RNA splicing, and cytoskeletal organization. In contrast, down-regulated genes such as DNPH1, EXOSC5, L3MBTL2, LGALS3BP, and SPRYD4 reflect impairments in nucleotide metabolism, RNA degradation, chromatin compaction, and intercellular communication, processes that are frequently disrupted during aging [54]. Similar transcriptional signatures have also been observed in human iPSC-derived cardiac progenitor cells cultured aboard the International Space Station, including upregulation of cell cycle regulators (CCND1, CCND2), the proliferation-associated growth factor (IGF2), and the cardiac differentiation marker (TBX3), accompanied by downregulation of extracellular matrix genes. These changes suggest a shift toward increased proliferation and structural remodeling [55]. Engineered human heart tissues exposed to long-term microgravity similarly displayed downregulation of contractile and calcium signaling genes, alongside increased expression of genes related to oxidative stress, mitochondrial dysfunction, and inflammation, consistent with aging-associated cardiac decline [56]. Additional evidence from single-cell RNA sequencing of immune cells revealed altered expression of genes involved in cytoskeletal organization, IL-6 signaling, and sirtuin-regulated metabolic control, suggesting disruption of immune homeostasis and activation of inflammaging-related pathways [57].

Collectively, these findings define a core set of microgravity-regulated genes in human cells whose altered expression mirrors aging-related molecular deterioration. Their functional roles in key cellular pathways highlight their potential as biomarkers of microgravity adaptation and as therapeutic targets for promoting resilience in aging tissues (Table 2). 

### 1.8. The International Space Station (ISS)

The International Space Station (ISS) is the largest scientific and technological international cooperative program worldwide. It represents a collaboration among multiple space agencies, including NASA (USA), the Canadian Space Agency (CSA), the European Space Agency (ESA), Roscosmos (Russia), and the Japan Aerospace Exploration Agency (JAXA) [58]. The ISS provides a unique environment for conducting microgravity research, offering insights into biological processes that are not possible under normal Earth conditions. Microgravity impacts biological organization at all levels, from cellular structures to whole organisms, and presents opportunities to explore novel pathways for therapeutic and pharmaceutical advancements. Experiments conducted in this environment can enhance our understanding of protein structures, gene expression, and potential drug and vaccine targets. The detrimental effects of aging and the physiological challenges posed by prolonged microgravity exposure make this environment particularly valuable for studying anti-aging therapies. Recent advancements in microgravity research aim to translate these findings into clinical applications and new drug developments. Platforms designed for autonomous and remote operations in microgravity contribute valuable data that informs both space exploration and health-related innovations on Earth [59]. However, it is important to acknowledge that microgravity is not the only environmental factor influencing biological systems aboard the ISS. Spaceflight exposes living organisms to a complex combination of stressors, including cosmic radiation, isolation, altered circadian rhythms, and psychological stress, all of which can interact with microgravity to shape biological and physiological responses. These combined factors distinguish true spaceflight experiments from ground-based microgravity simulations, such as the Random Positioning Machine (RPM) or Rotating Wall Vessel (RWV), which replicate the mechanical unloading aspect of microgravity but lack other space-specific stressors [52,59].

Microgravity-based studies have also paved the way for advancements in biotechnology, including the development of Organ-on-a-Chip (OOC) technology that involves microfluidic devices designed to replicate the architecture and microenvironment of tissues and organs, enabling more realistic physiological responses compared to traditional 2D or 3D culture systems [60]. These devices allow for customization specific to various diseases, making them particularly valuable for preclinical studies that account for cross-species differences. OOC platforms enhance drug screening processes by improving the predictability of efficacy, toxicity, and pharmacokinetics in humans, bridging the gap between in vitro assays and model studies [61]. Additionally, OOC technologies facilitate stratified medicine and the development of treatments for rare diseases and nanomedicine [62,63]. Utilizing OOC models in space can accelerate studies that would take years on Earth, mimicking drug effects on biological changes and reducing the need for animal studies in toxicity research [64].

### 1.9. Microgravity Research: Biological Application

Recent microgravity studies have further demonstrated that biological systems are profoundly impacted in space, providing valuable insights that are especially significant for aging and disease research. Monoclonal antibodies represent a promising strategy to selectively target key aging mechanisms, such as cellular senescence, inflammaging, and immunosenescence, thereby offering novel therapeutic avenues for managing cancer and chronic age-related disorders [41]. These therapeutics are increasingly used in modern medicine due to their high specificity and effectiveness in treating a range of age-associated conditions. Notably, microgravity has been shown to enhance the development of monoclonal antibodies, particularly by improving their crystallization, which facilitates more accurate structural analysis and better therapeutic design. This phenomenon not only demonstrates the unique capabilities of the space environment to influence biological systems but also underscores its relevance in exploring therapeutics aimed at addressing age-related conditions [65].

In parallel, microgravity influences cellular behavior, including changes in morphology, proliferation, and adhesion. A review published in *Frontiers in Cell and Developmental Biology* [47] found that cells exposed to microgravity undergo structural changes, affecting their function. These changes impact gene expression, protein synthesis, and cellular signaling, providing insights into cellular processes that cannot be fully understood on Earth [47]. Microgravity’s ability to affect biological systems extends into the realm of aging research. For instance, MRI scans of astronauts have revealed accelerated changes in brain white matter [51], and studies of immune cells indicate altered activity similar to that seen in aging. These findings suggest that examining aging in microgravity can provide new approaches to boosting immunity in older adults, capitalizing on the accelerated aging-like processes that occur in space. In addition, microgravity research has revealed how microgravity induces physiological changes similar to those seen in aging in the human tissues. One study demonstrated that simulated microgravity accelerated aging in human skeletal muscle myoblasts cultured in vitro. The research observed that exposure to microgravity led to decreased cell proliferation and increased cell size, similar to changes observed in aged muscle cells. These findings suggest that microgravity can accelerate muscle aging [48]. Another study investigated the impact of simulated microgravity on the carotid artery, revealing that exposure led to increased stiffness, thickness, and fibrosis, which are characteristic of vascular aging. The research also noted elevated senescence biomarkers in the carotid artery following simulated microgravity exposure [49].

This expanding research indicates that examining aging in microgravity may yield innovative treatment approaches for age-related diseases, capitalizing on the unique ability of the space environment to accelerate and amplify essential aging processes.

## 2. Discussion

Traditional Earth-based models have been essential in uncovering fundamental aging mechanisms, yet they often fall short in fully capturing the complexity and variability of age-related processes across different biological systems [66]. These limitations arise from species-specific differences, the inability to replicate certain physiological stressors, and the challenges inherent in modeling long-term aging processes in controlled settings [66]. The emergence of microgravity as a research tool provides a unique platform for investigating aging mechanisms in a controlled environment where these processes occur at an accelerated rate [53]. Studies have demonstrated that exposure to microgravity induces molecular and cellular changes that closely mirror the hallmarks of aging [1], including genomic instability, telomere shortening, and cellular senescence. These findings suggest that microgravity not only accelerates aging-related changes but also enables the dissection of underlying mechanisms within a significantly shorter timeframe, offering unprecedented opportunities for aging research.

Studies conducted on various human cell types exposed to microgravity have demonstrated consistent gene expression changes that parallel key molecular features of aging. These findings support the concept that microgravity induces molecular and cellular alterations resembling aging-associated decline, including disruptions in chromatin remodeling, mitochondrial function, cytoskeletal dynamics, and immune regulation [54,55,56,57]. This growing body of evidence validates microgravity as a relevant and accelerated model for aging research and highlights its potential to uncover novel therapeutic targets to mitigate age-related dysfunction.

In addition to advancing fundamental aging research, microgravity holds therapeutic potential for addressing age-related degeneration. Cellular and tissue models exposed to microgravity have exhibited enhanced regenerative properties, improved stem cell proliferation, and three-dimensional tissue organization, all of which are crucial for developing regenerative medicine strategies [67,68]. These effects highlight microgravity’s potential as a novel therapeutic modality for age-related diseases, including osteoporosis, sarcopenia, and neurodegenerative disorders [69]. The ability to manipulate these cellular responses in microgravity offers an opportunity to develop targeted interventions that could ultimately translate into clinical applications on Earth.

It is important to note that exposure to microgravity is already widely implemented in both research and commercial contexts, including through parabolic flights used for scientific studies, astronaut training, and space tourism preparation. These platforms have supported biological and medical research with standard ethical oversight and without raising significant ethical controversies to date, particularly when participation is voluntary [70,71,72].

While microgravity holds significant promise for aging research and therapy, several challenges must be addressed. The long-term biological effects of microgravity exposure require further investigation, particularly in terms of adaptation and the reversibility of observed changes [73]. Moreover, translating microgravity-based discoveries into effective terrestrial therapies demands scalable and cost-efficient strategies, highlighting the need for space-based biotechnologies that can seamlessly integrate with conventional biomedical research. Overcoming these challenges will be essential for fully leveraging microgravity’s potential in aging studies and therapeutic advancements.

In light of these limitations, researchers are increasingly turning to advanced microgravity platforms that enable more precise and complex biological investigations. As space-based technologies continue to evolve, the potential to conduct complex, long-term experiments in microgravity becomes increasingly attainable. Platforms such as the ISS and emerging commercial space stations [58,59] allow for the integration of Organ-on-a-Chip systems with real-time monitoring tools, enabling precise analysis of aging-related processes over time [60]. By combining these systems with omics technologies, such as transcriptomics, proteomics, and metabolomics, researchers can capture a comprehensive picture of how aging unfolds at multiple biological levels in microgravity. This integrated approach may reveal molecular pathways that are either altered or show aging-like changes in response to microgravity, offering insights that are not captured by traditional Earth-based aging models.

Hence, despite these challenges, microgravity offers a groundbreaking approach to studying aging and longevity. By accelerating biological processes and facilitating innovative experimental strategies, it enhances our understanding of the molecular mechanisms underlying aging while paving the way for novel therapeutic developments. As research progresses, microgravity may prove to be a valuable tool not only for investigating aging but also for developing interventions that extend health span and longevity on Earth.

## Figures and Tables

**Table 1:** Major aging treatments and their associated effects.

Treatment/Approach	Therapeutic Category	Associated Effect	Reference
Rapamycin	Pharmacological	mTOR inhibitor enhanced resilience	[29]
Senolytics (Dasatinib, Quercetin)	Pharmacological	Reduced senescence, improved tissue function	[32]
Metformin	Pharmacological	Improved mitochondrial function, reduced oxidative stress, and inflammation	[38]
Resveratrol	Pharmacological	Antioxidant effects, activation of longevity-associated genes	[38]
Caloric restriction	Non- Pharmacological	Reduced inflammation, improved metabolic health	[39]
Physical exercise	Non- Pharmacological	Enhanced metabolic, cardiovascular health	[39]
Stem cell therapies	Biologics	Tissue regeneration, immune modulation	[40]
Monoclonal antibodies (anti-senescence)	Biologics	Targeting senescent cells, modulation of age-related pathways	[41]
Biomaterials, scaffolds	Materials	Tissue engineering	[42]

**Table 2:** Genes regulated by microgravity across human cell types and their associated functions.

Gene Symbol	Regulation	Associated Function	Reference
CSGALNACT2	Upregulated	Biosynthesis, inflammation response	[54]
CSNK2A2	Upregulated	Cell cycle regulation, DNA repair, and apoptosis	[54]
HIPK1	Upregulated	Apoptosis, development, and stress response pathways	[54]
MBNL2	Upregulated	RNA-binding protein, alternative splicing regulation	[54]
PHF21A	Upregulated	Transcriptional repression, chromatin remodeling	[54]
RAP1A	Upregulated	Cell adhesion, proliferation, and differentiation	[54]
DNPH1	Downregulated	Catabolism of deoxynucleotides	[54]
EXOSC5	Downregulated	RNA processing	[54]
L3MBTL2	Downregulated	Regulation of gene expression, chromatin organization	[54]
LGALS3BP	Downregulated	Immune response, cell adhesion	[54]
SPRYD4	Downregulated	Cell cycle regulation	[54]
CCND1	Upregulated	Cell cycle, cell proliferation	[55]
CCND2	Upregulated	Cell cycle, cell proliferation	[55]
IGF2	Upregulated	Cell cycle, cell proliferation	[55]
TBX3	Upregulated	Cardiac differentiation	[55]
Contractile and calcium genes	Downregulated	Muscle contraction, calcium handling	[56]
Oxidative stress and inflammatory genes	Upregulated	Inflammation, mitochondrial dysfunction	[56]
IL-6 pathway genes	Altered	Inflammation, immune regulation	[57]
Sirtuin-regulated genes	Altered	Metabolic control, aging resilience	[57]

## Abbreviations

ISS	International Space Station
ROS	Reactive oxygen species
SASP	Senescence-associated secretory phenotype
CVD	Cardiovascular diseases
AD	Alzheimer’s disease
mTOR	mechanistic target of rapamycin
OOC	Organ-on-a-Chip
